# Crop Mass and N Status as Prerequisite Covariables for Unraveling Nitrogen Use Efficiency across Genotype-by-Environment-by-Management Scenarios: A Review

**DOI:** 10.3390/plants9101309

**Published:** 2020-10-02

**Authors:** Gilles Lemaire, Ignacio Ciampitti

**Affiliations:** 1 Department Environment & Agronomy, INRA, 86600 Lusignan, France; 2 Department of Agronomy, Kansas State University, Manhattan, KS 66506, USA

**Keywords:** nitrogen use efficiency, nitrogen nutrition index, genotype-by-environment interactions, critical N uptake, maize, sorghum

## Abstract

Due to the asymptotic nature of the crop yield response curve to fertilizer N supply, the nitrogen use efficiency (NUE, yield per unit of fertilizer applied) of crops declines as the crop N nutrition becomes less limiting. Therefore, it is difficult to directly compare the NUE of crops according to genotype-by-environment-by-management interactions in the absence of any indication of crop N status. The determination of the nitrogen nutrition index (NNI) allows the estimation of crop N status independently of the N fertilizer application rate. Moreover, the theory of N dilution in crops indicates clearly that crop N uptake is coregulated by (i) soil N availability and (ii) plant growth rate capacity. Thus, according to genotype-by-environment-by-management interactions leading to variation in potential plant growth capacity, N demand for a given soil N supply condition would be different; consequently, the NUE of the crop would be dissimilar. We demonstrate that NUE depends on the crop potential growth rate and N status defined by the crop NNI. Thus, providing proper context to NUE changes needs to be achieved by considering comparisons with similar crop mass and NNI to avoid any misinterpretation. The latter needs to be considered not only when analyzing genotype-by-environment-by-management interactions for NUE but for other resource use efficiency inputs such as water use efficiency (colimitation N–water) under field conditions.

## 1. Introduction

Globally, nitrogen (N) is a critical factor limiting agricultural productivity, in addition to the supply of water and other nutrients such as phosphorus. For the last five decades, the external application of mineral N fertilizers increased sevenfold while agricultural food production only doubled [1]. Therefore, in order to meet the demand of the overgrowing human population, the utilization of mineral N fertilizers is one of the key factors [2,3]. Nevertheless, mineral N fertilizers are provided via the industrial chemical reduction of atmospheric N, which is associated with substantial greenhouse-gas emissions. Moreover, for intensive agricultural systems, a large amount of mineral N supply produces severe environmental effects such as eutrophication of freshwater [4] and marine ecosystems [5], groundwater pollution, and greenhouse-gas emissions (e.g., N oxides and ammonia) [6,7].

The objective of sustainable N fertilization management should be to increase the synchrony between crop N supply and crop N demand in order to constrain N losses. Consequently, the conventional method using an analysis of the yield response to the application of mineral N fertilizer, with the goal of defining the “optimum” N availability for the crop to achieve a target yield, becomes inefficient. Due to large uncertainties in the estimation of (i) the quantity of available N supplied by soil, and (ii) the crop N demand associated with the attainable yield in various conditions (related to soil and weather variations), crop fertilization management often leads to excess application rates due to risk aversion by farmers of not matching crop N demand [8]. Thus, it is clear that a change in paradigm is needed in order to adopt a method involving the quantification of plant N demand using a more dynamic approach [9] and the monitoring of N supply to match crop demand. Consequently, the current questions dealing with sustainable development, climate change, quality of environment, and food security are strongly associated with the use efficiency of N fertilizers in the current farming systems [10].

## 2. Nitrogen Use Efficiency by Crops and Its Components

Improving N use efficiency (NUE, herein defined as the increase in yield per unit of fertilizer applied) is a major goal in plant breeding for sustainable agriculture [11]; a crop with greater NUE should reach a given target yield with reduced fertilizer N supply, which should decrease its environmental impact [4,5,6,7,12].

**Figure 1:**
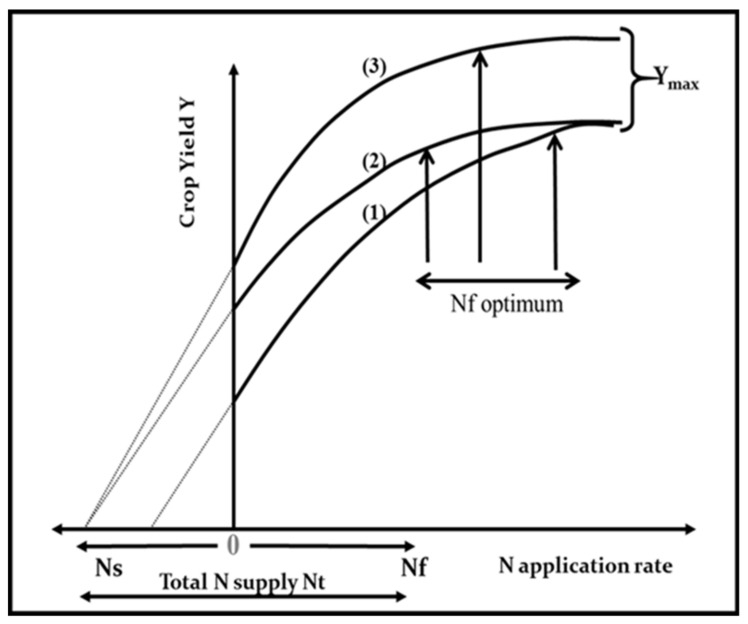
Representation of differences among the response of crop yield (Y) to N fertilizer application rates (N_f_) according to variations in (1) soil N supply (Ns), (2) yield without external N application, and (3) maximum yield (Y_max_). All these variations lead to differences in optimum N fertilizer application rate. Note: the Nf optimum is arbitrarily placed at the onset of the plateau region for the yield-to-N-supply response curve.

In a more formal definition, the NUE of a crop is its capacity to increase yield (∆Y) per unit of N added via fertilization (∆N_f_) (NUE = ∆Y/∆N_f_). Then, the NUE can be calculated from the derivative of the response curve for yield to the total N supply (Figure 1). Owing to the asymptotic nature of this response curve, NUE declines as N supply increases. According to variations in soil N supply due to local climate and soil conditions, previous crop type, and management, important differences in NUE can be achieved at similar rates of N fertilizer supply. Therefore, a comparison of the NUE among crops (different species of cultivars) must be done within the same range of N supply. Additionally, for understanding the contribution of both soil and plant processes, Moll et al. [13] offered to dissect NUE as the product of two individual components:

(i) the N uptake efficiency, referring to the increase in plant N uptake per unit of N supply (N_f_), also termed the N recovery efficiency (NRE),
(ii) the N conversion efficiency (NCE), described as the crop’s ability to produce an increment in biomass per unit of N taken up by the plant.

Yield represents either the aboveground biomass, as for forage crops, or the grain mass, as for cereals, grain legumes, or oil seed crops. For grain crops, harvest index (HI) should be taken into account as a relevant component for NUE. Thus, NUE can be formulated as follows (Equations (1) and (2)):

(1) $$\mathrm{NUE}=\frac{\Delta \mathrm{Nupt}}{\Delta \mathrm{Nf}}*\frac{\Delta W}{\Delta \mathrm{Nupt}}*\frac{\Delta Y}{\Delta W}$$

(2) NUE=NRE∗NCE∗HI

Therefore, NUE results from three types of processes: (i) the ability of the crop to capture N (Nupt); (ii) the competence of the crop to utilize N for biomass (W) production; (iii) the capacity of the crop to allocate C and N to reproductive organs (e.g., grains in maize) for yield (Y) formation.

## 3. The Coregulation of N Uptake by Soil N Supply and Plant Growth (N Demand)

Crop N demand (N_c_ expressed in kg·ha^−1^) or critical N uptake can be defined as the quantity of total crop N uptake corresponding to the maximum biomass achievable in a given environmental condition without any limitation of N (W_c_ expressed in t·ha^−1^). Then, N_c_ can be determined by the product of W_c_ and the critical plant N concentration (%N_c_), i.e., the minimum %N_c_ for maximum plant growth rate [9] (Equation (3)).

N_c_ = %N_c_ W_c_. (3)

Greenwood et al. [9] showed that %N_c_ declines as crop biomass increases, resulting in the so-called N dilution process. In addition, the same authors [9] showed that C4 species differentiated from C3 by having a lower value of coefficient a_c_ according to their metabolic difference. Past studies with a broad range of crops already demonstrated that the decline in %N_c_ with W_c_ can be represented by a unique negative allometric function for a given species [14,15](Equation (4)).

%N_c_ = a_c_ (W_c_)^−b^, (4)

where coefficient a_c_ represents the value of %N_c_ for W_c_ = 1 t·ha^−1^, and coefficient b is dimensionless.

Combining Equations (3) and (4) gives the dynamics of the crop N demand, N_c_, in relation to the dynamics of potential crop biomass accumulation, W_c_. Then, crop N demand, N_c_, is defined as the minimum crop N uptake necessary for reaching maximum crop biomass accumulation (Equation (5)).

N_c_ = a_c_′ (W_c_)^1 − b^, (5)

where coefficient a_c_′ refers to the plant N demand for a potential biomass of 1 t·ha^−1^. The value of a_c_′ equals to 10a_c_, when a_c_ is expressed in g N·100 g dry matter and a_c_′ is expressed in kg N·ha^−1^. Values of coefficients a_c_ and b have been determined empirically for several crop species [14]. As demonstrated in Lemaire et al. (2008) [14], Equation (5) allows the determination of the N nutrition status of a given crop by calculating the relative distance of the data point N_act_–W_act_ (where N_act_ and W_act_ are the observed value of N uptake and crop mass in a given condition) to the “critical N uptake curve” (Equation (6)).

NNI = N_act_/N_c_, (6)

where Nc is the “critical N uptake”, corresponding to a value of W_c_ = W_act_ in Equation (5). Therefore, a value of NNI above 1 would indicate a luxury N uptake, while a value of NNI below 1 would indicate an N deficiency and allows its quantification; an NNI of 0.6 indicates that, at the time of the estimation, the crop has taken up only 60% of its N demand as determined by its actual biomass.

Equation (5) can be derived from expressing the crop demand in terms of N uptake by unit of crop mass dN/dW (Equation (7)).

dN/dW = a′_c_ (1 − b)W_c_^−b^. (7)

Then, the crop N demand can be expressed in dynamic terms as follows (Equation (8)):

(8) $$\frac{\mathrm{dNc}}{\mathrm{dt}}=\frac{\mathrm{dNc}}{\mathrm{dWc}}\frac{\mathrm{dWc}}{\mathrm{dt}}=a^{\prime }{}_{c}(1-b)Wc^{-b}\left(\frac{\mathrm{dWc}}{\mathrm{dt}}\right)$$

Crop N demand rate (kg N·ha^−1^·day^−1^) is determined by the crop growth rate (t dry matter·ha^−1^·day^−1^); however, as crop mass increases, the proportionality coefficient between daily crop N demand and daily crop mass increment declines (Equation (8)). When soil N availability becomes limiting, the plant is not able to satisfy its own N demand and then cannot follow the critical N–W trajectory (Equation (5)), thus following a lower curve with a coefficient a′f < a′c, whose value depends on the level of soil N availability (Equation (9)).

N = a′_f_ W^1 − b^. (9)

The value of a′_f_ reflects the N nutrition status of the crop for a steady-state level of N soil availability as a′_f_/a′_c_ = NNI. Coefficient a′_f_ denotes then the regulation of N uptake by soil N availability and then depends on the N supply conditions that determine the N–W trajectories for each N treatment (Equation (9)). However, according to unknown soil N supply, the correspondence between N fertilizer and application rate is ambiguous. This coregulation of crop N uptake by both soil N availability and plant potential growth rate was already formulated and analyzed experimentally on wheat [16]. The regulation of the root N absorption capacity of plants by shoot signals is now very well documented at molecular physiology levels (see [17] for a review). This regulation implies a feedback stimulation of root N absorption linked with leaf photosynthesis activity [18,19], and a feedback repression linked with shoot N satiety signals [20,21]. These integrated signals regulate root N acquisition for harmonizing plant N demand, mainly determined by its growth rate [21], and they explain the strong proportional relationship between N and W^1 − b^. The value of coefficient 1 − b was demonstrated to be close to 2/3 for a large range of species because N scales with plant area (light interception and photosynthesis), while W scales with plant volume [22].

**Figure 2:**
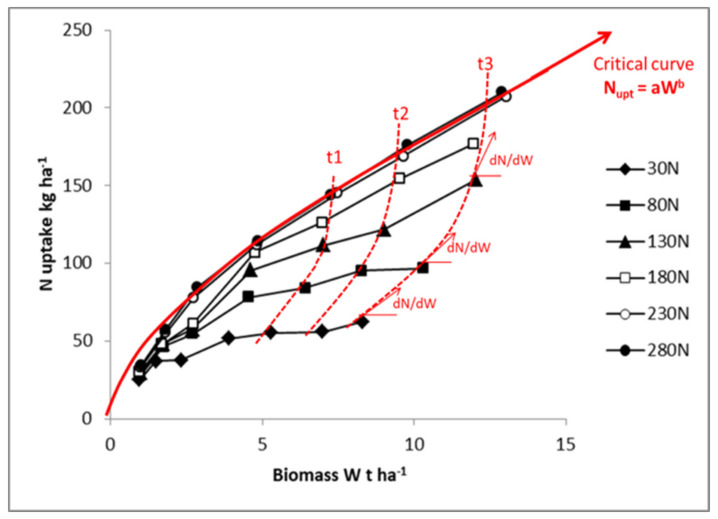
Plant N uptake relationship with relative crop biomass for maize crops receiving different N fertilizer application rates (from 30 to 280 kg N·ha^−1^). The red full line represents the critical N uptake curve for maize (N_c_ = 34W_c_^0.64^). The dotted lines denote the inverse of the response of W to N uptake at different time (t1, t2, t3, …), and dN/dW represents the inverse of N conversion efficiency. Different symbols represent the N fertilizer application rates. Data redrawn from Plénet and Lemaire [22].

**Figure 3:**
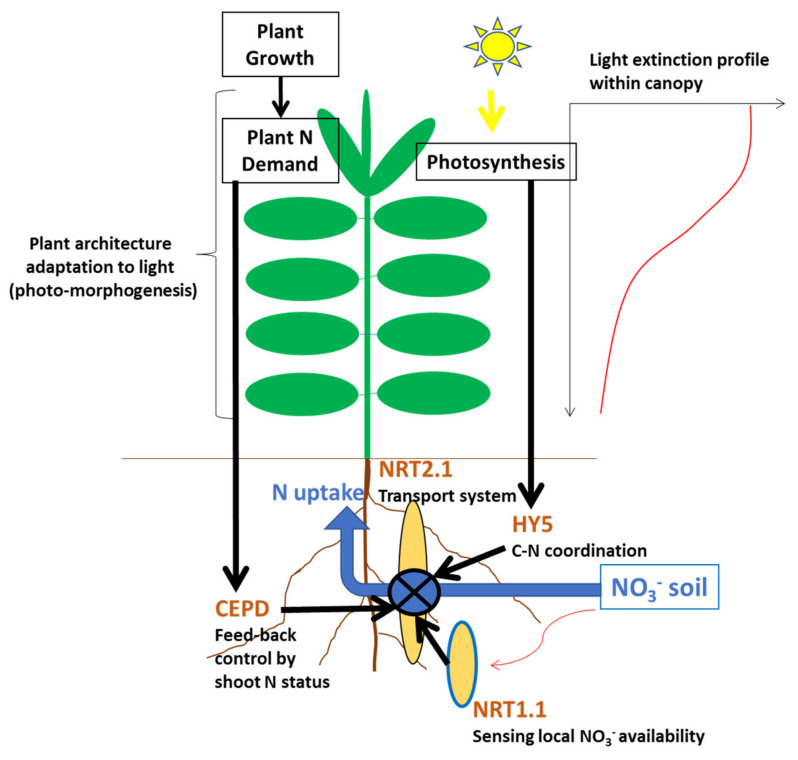
Schematic representation of N uptake regulation system of a plant growing within a dense crop. The regulation of the expression of gene NRT1.1 encoding the transport of nitrate via root plasma membrane is represented by dark arrows and the identification of corresponding molecules: (**i**) NRT1.2, a sensor of local NO_3_^−^ availability in soil; (**ii**) C-terminally encoded peptide downstream (CEPD), a gluta-redoxine protein responsible for feedback control by plant N demand through N satiety signals; (**iii**) HY5, a transcription factor for the coordination between N and C acquisition by plants. For more details on this molecular network, please see the review of Briat et al. (2020) [17]. The environmental constraint in which plants are situated within a canopy is represented by the light profile extinction and the mention of plant architecture. For example, the ratio between metabolic tissues associated with leaf area and structural tissues associated with stem, petioles, and midribs is highly determined by the shade avoidance adaptation of plant through photo-morphogenesis.

The coregulation of crop N uptake by both soil N supply and plant N demand, as expressed in Equation (8) and represented in Figure 2, is achieved through a network of physiological and metabolic processes at whole plant and canopy scale. The dependency of plant N uptake from both NO_3_^−^ and NH_4_^+^ concentration in soil solution is expressed by the value of coefficient a′_f_ in Equation (8). The plant N response to the uptake of NO_3_^−^ or NH_4_^+^ is regulated by the activity of corresponding transport proteins in the plasma membrane of root cells: high-affinity or low-affinity transport systems (HATS and LATS, respectively) [20,23]. It is possible to link plant N uptake capacity to (i) the density of transport proteins per unit of root length, and/or (ii) the intrinsic activity of these transports systems. However, these N transport systems are very plastic because the expression of these types of genes is highly affected by plant N demand associated with the whole plant growth [20]. Three types of regulation have been identified at the plant scale: (i) a local and rapid (few hours) stimulation, corresponding to a fast dose-dependent increase in N transport activity following NO_3_^−^ provision to root [24]; (ii) a longer-term (few days) feedback repression of root N uptake systems associated with shoot N satiety signals [20,25,26] that modulates root N absorption for matching the N demand of the whole plant as determined by its growth rate [27]; (iii) an upregulation of root N transporters by signals from photosynthesis also participates in the control of plant growth on N uptake [18,25,28] via regulation of N–carbon (C) acquisition. All these coordinated molecular processes explain the reason why the dynamic of N uptake by crops appears so precisely linked to the crop biomass accumulation dynamic, as illustrated in Figure 3. The same type of regulation was documented for NH_4_^+^ absorption [29], as well as analogous feedback control of N_2_ symbiotic fixation for legumes [30].

Why is plant N uptake proportional to W^1 − b^ and not to W? A strict proportionality of N to W would lead to a constant N concentration in the plant, which is obviously not the case. Therefore, the main question to be asked is the following: What is the main reason that plants are unable to maintain a constant N concentration in their tissue (homeostasis) when crop mass increases? Lemaire and Gastal [15] showed that, even when plants are growing in isolation, plant %N declines as plant mass increases, but this N dilution as expressed by Equation (4) is relatively low (b = 0.10–0.15) as compared to the more rapid dilution observed for the same plants in a dense canopy (b = 0.33). This dilution in isolated plants has been interpreted as the necessity for a plant to invest a minimum of biomass in supporting tissues (such as vasculary bundles, sclerenchyma, and collenchyma having low N content) to maintain their metabolic tissue (leaf parenchyma) as plant size increases [31]. When these plants are growing in a dense canopy, competition for light with their neighbors requires them to invest a greater proportion of their biomass in supporting structural tissues (having low N concentration) for positioning their leaf area within the well-illuminated layers of the canopy [32]. Moreover, plants allocate N preferentially to the well-illuminated leaf layers, and the N of shaded leaves is recycled for new leaf area expansion at the top of the canopy, accelerating the N dilution process. Therefore, the N dilution process in plants growing in a dense canopy is the consequence of the two adaptive mechanisms to competition for light: (i) a shade avoidance mechanisms determined by the photo-morphogenetic response of plants to changes in light quality associated with a light extinction profile within the canopy [33,34], and (ii) an optimization of N allocation within the plant to well-illuminated leaf layers for maximizing radiation use efficiency [35,36,37].

This short overview of coregulation of N uptake by both soil N supply and plant growth capacity demonstrates the necessity to establish bridges between “plant physiology and metabolism” and “field crop ecophysiology” approaches. Reductionist analysis allows the identification of molecular processes involved in plant N nutrition regulation; however, without any functional frame describing the real set of constraints to which an individual plant has to adapt within a dense canopy, the relevance or the accuracy of these molecular processes is impossible to establish. Reciprocally, allometries observed at whole plant and canopy scales must be based on the reality of molecular processes, not simply as a description, but as an expression of the emergent properties of the whole plant–environment system.

## 4. Crop Mass Accumulation Drives Both NCE and NRE

High potential crop growth rate, due to coregulation of factors related to genotype and/or favorable environment, would present greater N uptake rate than a crop displaying a lower potential growth rate. Thus, following Equation (6) and Figure 2, this crop should have superior NCE because NCE increases (or dN/dW decreases) as W increases. Then, when one compares crops having different mass, the bigger crop would have always a higher NCE because its marginal N demand per unit of biomass declines as W increases. Consequently, comparisons of NCE across species, cultivars, or environments must be done only with similar crop mass. Otherwise, the result would demonstrate that a bigger plant gives rise to the highest NCE, which is a trivial result.

**Figure 4:**
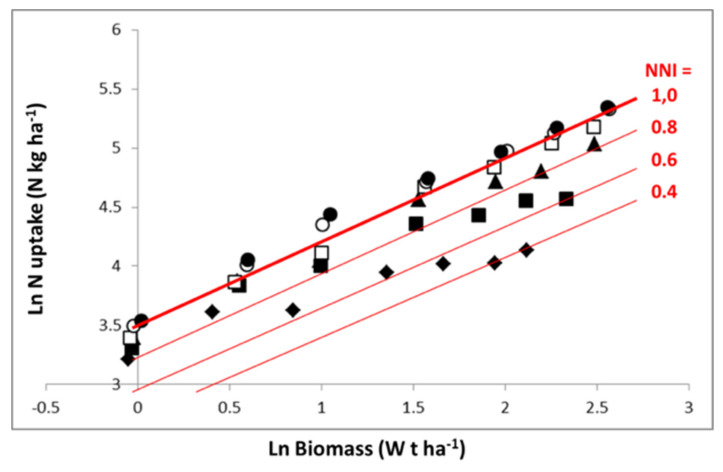
Representation of data of Figure 2 on a log–log scale (similar symbols). The thick red line denotes the critical N uptake curve for maize established by Plénet and Lemaire [22], LnN = 3.5 + 0.64 LnW, and the thin red lines denote the N–W trajectories corresponding to limiting and constant N availability with nitrogen nutrition index (NNI) of 0.8, 0.6, or 0.4. Symbols are the same as in Figure 2, representing different levels of N fertilizer application rates.

Representation in Figure 4 in log–log scale allows an easier analysis of the N–W dynamics according to N fertilization rate. Equation (7) is then transformed into a linear one. For the highest N application rate, the crop follows an LnN–LnW trajectory close to the “critical curve”, for just a short period with N in excess. For lower N application rates, the crop starts with a relatively low N deficiency (1 > NNI > 0.8) and progressively experiences a more pronounced N deficiency as soil N availability is exhausted, leading to an NNI of 0.6 and 0.4 for the application rates of 80 kg N·ha^−1^ and 30 kg N·ha^−1^, respectively. Therefore, the trajectories followed by crops under different N supply conditions are the result of (i) dynamics of crop mass accumulation as determined by W^1 − b^, and (ii) the fluctuation of coefficient a′_f_(t) with time, reflecting variations in soil N availability according to soil N net mineralization and plant N absorption.

It is clear that the interpretation of differences in NCE observed across crop species, cultivars, or environment–management conditions must be interpreted in terms of not only the differences observed in crop mass, but also the differences observed in NNI.

**Figure 5:**
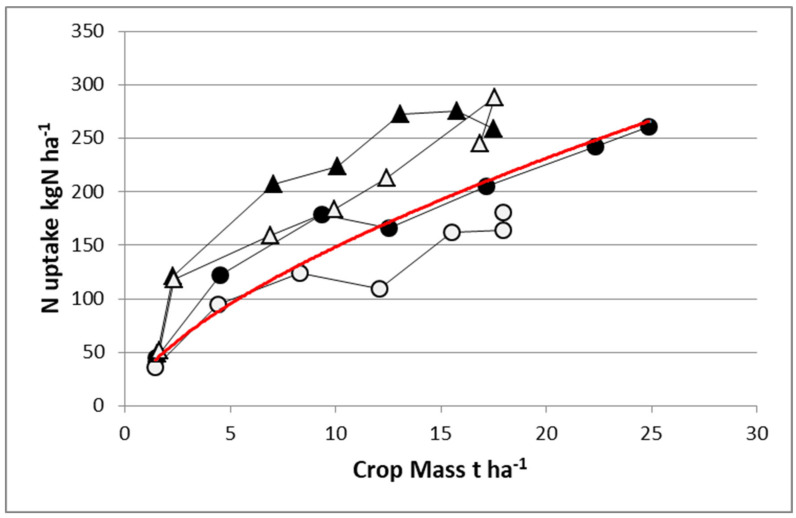
Crop N uptake and aboveground biomass (W) for maize (circles) and sorghum (triangles) growing with an N fertilizer application rate of 200 (dark) or 30 (open) kg N·ha^−1^. The red line denotes the “critical N uptake curve” common for both crops: N = 34 W^0.64^. Redrawn from Lemaire et al. 1996 [38].

What is more complicated is that the NNI at which a crop is able to maintain with a given N supply condition, i.e., the position of its N–W trajectory vis-à-vis the “critical N–W curve”, depends highly on its own efficiency for taking up soil N (as reflected by its NRE). Thus, with a given N supply condition, species or cultivars having a superior NRE would be expected to conserve a greater NNI value than those having a lower NRE. Thus, classification of NNI reached by species and genotypes cultivated in the same N supply condition should reflect their differences in NRE. Figure 5 shows the difference in the same experiment between maize and sorghum under irrigation with two contrasting N fertilization levels. With a high N fertilizer supply, sorghum accumulated the same quantity of N in shoots as maize (275 kg N·ha^−1^) despite achieving a reduced biomass (17.5 vs. 25 t·ha^−1^). Hence, sorghum was in large excess of N nutrition (NNI = 1.38), while maize was just nonlimiting (NNI = 0.99). With a limiting N application rate, sorghum remained able to uptake 250 kg N·ha^−1^ and to maintain an NNI = 1.22, while maize was able to uptake only 175 kg N·ha^−1^, which led to a strong N deficiency (NNI = 0.78). It is clear that sorghum shows a much higher NRE than maize, maintaining a nonlimiting N status even with very low N application rate.

Analyzing the NUE between species, we may conclude that NUE for sorghum is very low due to the low level of biomass response to N fertilization relative to the large response displayed by maize (W of 7 t·ha^−1^). This example clearly demonstrates a trade-off between NCE and NRE (as components of NUE); species or cultivars with a high NRE have a greater NNI than their counterparts having a low NRE, leading to a lower apparent NCE. Clearly, this case study reinforces the need for simultaneously analyzing both NRE and NCE for NUE when comparing within or between crops, and results should be interpreted through the examination of NNI and crop mass as covariables for avoiding misinterpretations.

Variations in N uptake across genotypes were evaluated in rice [39], wheat [40], and maize [41]. Other studies demonstrated that the N uptake capacity of modern cultivars is greater than that of older ones in relation to their increased biomass accumulation [42,43]. This result illustrates the coregulation of plant N uptake by soil N supply and plant growth (and plant N demand). As a consequence, breeding efforts for improving biomass accumulation in modern cultivars should result in superior NRE and NCE. A more interesting result would be to improve the intrinsic N uptake capacity, i.e., N uptake at similar crop mass. Some studies demonstrated differences in intrinsic N uptake capacity among species such as cocksfoot versus tall fescue [44] and sorghum versus maize [38].

Variations in the intrinsic ability of plant N uptake across genotypes within the same species are scarcely reported. One exception to this rule was documented by Sadras and Lemaire [45], displaying different N uptake at similar crop mass. Laperche et al. [46] identified quantitative trait loci (QTL) for root architecture co-locating with QTL for traits associated with N uptake efficiency in wheat. Furthermore, for root architecture (density and length of lateral roots), a large variation was detected across maize lines [47]. Therefore, it seems feasible to pursue different avenues for breeding genotypes with a higher N uptake under low N availability. Such a higher NRE would lead to a decrease in N required for a target yield. Genetic studies reported that NRE was the most important component of NUE in rice [48] and wheat [40].

**Figure 6:**
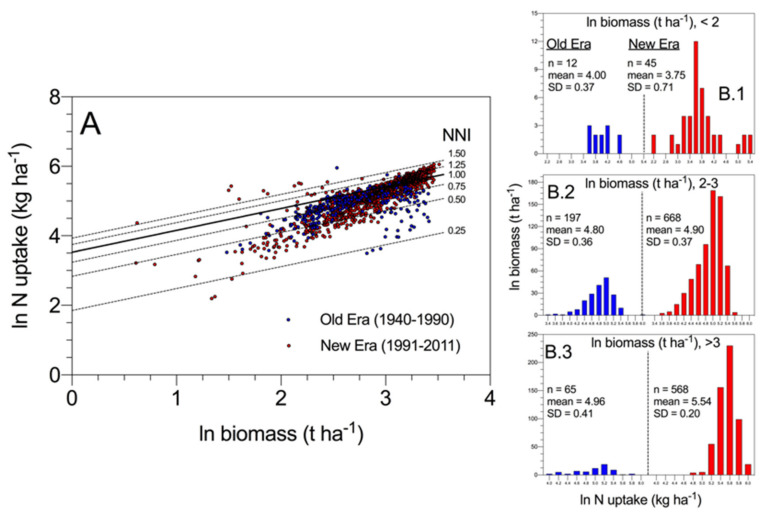
Panel (**A**) represents a log–log scale (similar symbols) of changes in N uptake and biomass for historical maize hybrids with respect to with their NNI level at maturity. Higher efficiency in the conversion of N uptake to yield is not necessarily connected to changes in N dynamics but to the ability of modern maize hybrids to increase plant growth and attainable yield. NNI = 1 refers to the calculation presented in Figure 2. Panels (**B.1**–**B.3**) represent data retrieved from Ciampitti and Vyn (2012) [49].

As shown in Figure 6, results obtained by Ciampitti and Vyn (2012) [49] revealed that most of the variation in N uptake across historical maize hybrids at maturity was associated with variations in crop mass accumulation and then crop N demand when comparing “new era” to “old era” cultivars, in addition to potential changes in intrinsic N uptake efficiency (i.e., at comparable biomass) (Figure 6A). When exploring the data with different ranges of biomass, N uptake increased more proportionally with biomass for modern relative to old maize hybrids (Figure 6B.1–B.3). These data provide for the first time for maize an indication that higher N uptake for the modern relative to older hybrids is not only due to the increase in biomass (average ln biomass 3.21 vs. 3.19 for modern vs. old hybrids, >3 ln biomass range, panel B.3). In addition, there is evidence that a genetic change in intrinsic N uptake efficiency (i.e., at comparable biomass) may have taken place primarily under a larger biomass level (>20 t·ha^−1^). Lastly, the narrower variation in N uptake for modern maize hybrids also reflects an overall improvement in the breeding selection process for excluding genotypes with lower NNI values.

Several mechanisms could be involved in breeding for plants with a higher capacity to take up N in limiting N supply conditions: (i) a dense and highly ramified root system architecture allowing a more efficient foraging and rapid absorption of soil mineral N, and a greater competitive ability against microbes; (ii) more efficient nitrate and ammonium channels across the root membrane, which would also contribute to a higher competitive ability of plants with soil microbes for mineral N released by gross mineralization; (iii) the quantity and the quality of root exudates which can regulate soil microbe community dynamics and, consequently, gross N fluxes in the rhizosphere [50].

These mechanisms operate concurrently in soils with multiple trade-offs. They are important keys for understanding and interpreting genotype-by-environment-by-management (G × E × M) interactions. However, the phenotypic variation in NRE observed across cultivars must be analyzed with a clear separation of the variation in N uptake directly linked to changes in crop mass from the intrinsic N uptake capacity of the crop at a similar mass level [51].

This difficulty in comparing NRE among genotypes could be overcome by ranking genotypes on the basis of their NNI values when N supply is limiting. In this way, it should be possible to rank genotypes according to their capacity to match their own N demand. Such an approach to comparing varieties under limiting N was used for analyzing the genetic improvement in Australian wheat cultivars [52], showing an increase in NNI with the year of cultivar release. Therefore, this result shows that breeding efforts for high yield can also improve the capacity of crops to take up N and then to maintain their N status in limiting N supply conditions. This study underlines the need to use the NNI approach for improving our comprehension of the NUE trait and its related soil–plant processes [45].

Two important consequences can be drawn from the above analysis:

(i) NRE and NCE increase both with plant mass as the result of the feedback control of N uptake by plant growth rate and its translation to the critical N dilution curve. The dependency of NRE upon crop growth capacity is a direct consequence of the coregulation of plant N uptake by soil N availability and plant growth. Thus, in a given soil supply condition, a crop having a higher growth rate should recover a greater proportion of soil N than a plant having a lower growth capacity. The dependency of NCE upon crop growth capacity is simply the expression that plant N demand per unit of crop mass (dN/dW) decreases allometrically with plant mass. Hence, a crop having a high biomass has higher NRE and NCE than a crop presenting low biomass. Hence, breeding for high crop mass should automatically lead to improved NUE.

(ii) It is more interesting to obtain genotypes having higher NUE at comparable mass. Owing to the very limited variation of the critical N dilution curves across species (except C3 vs. C4), there is low possibility to get variation in NCE at similar crop mass across genotypes within the same species. Thus, the possibility to increase NCE through plant breeding is very challenging, but potential changes in N allocation within the plant and N redistribution within the maize canopy profile could improve overall utilization efficiency. Increasing NCE could be achieved via two ways: (1) decreasing %N in the “metabolic compartment” while maintaining a similar photosynthesis activity per unit of N as for C4 vs. C3, and/or (2) decreasing %N in the “structural compartment”, which would correspond to a more efficient allocation of N to well-illuminated leaf areas within canopies, ultimately modifying the critical N dilution curve during crop growth and development. However, the large variation in NRE at similar crop mass existing across species would indicate that intraspecific variability should be large enough for justifying a breeding effort. In this way, ranking genotypes by their aptitude to maintain a high NNI in limiting N supply conditions should be a relevant breeding program. However, for grain crops, even if two components of NUE, NCE, and NRE have to be considered as important parameters, it is necessary to also consider a third component, i.e., the harvest index (HI).

## 5. Importance of Harvest Index for Nitrogen Use Efficiency

**Figure 7:**
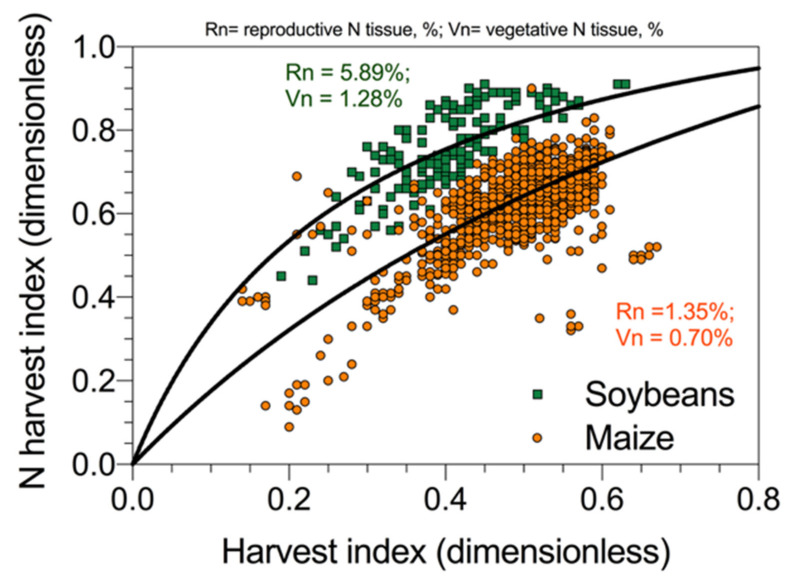
Relationship between nitrogen harvest index and dry mass harvest index for two crops, soybeans and maize, reporting the overall average N concentration for the reproductive (grain/seed) and vegetative (stover) plant fractions. Data redrawn from Ciampitti and Vyn (maize) [49] and from Tamagno et al. (soybeans) [55].

The harvest index (HI), i.e., the grain mass per unit of aboveground biomass, is an important component of NUE, and its variation across crop species and cultivars is an important objective for plant breeding. In addition, the N harvest index (NHI), i.e., N allocated to the reproductive organ relative to total plant N uptake, is an important feature for yield. The NHI determines the grain protein content [53], which is a determinant of grain nutritional quality. Desai and Bhatia [54] reported that NHI was correlated to HI for several durum wheat cultivars. Tamagno et al. [55] also demonstrated the same concept for soybeans following a model of N allocation between vegetative and reproductive plant fractions as first introduced by Lawn [56]. A representation of the relationship reported in soybeans [55] and maize [49] is presented in Figure 7. This result indicates that the partitioning of N between vegetative and reproductive organs follows more or less the allocation of dry matter. However, in legumes (e.g., soybeans) given the right N demand in seeds relative to the rest of the plant, the proportion of N allocation exceeds that for dry mass, while, in cereals (e.g., maize), the allocation of N and mass is more proportional (Figure 7). Therefore, the relationship between NHI and HI is a direct effect of N dilution (allocation) in the reproductive organs, with increments in mass obtained with a reduced N/mass ratio, with larger differences between NHI and HI for crops with high protein levels. Grain yield per unit of N uptake and grain/seed N concentration are inversely related [57]; then, for different crop species, cereals, grain legumes, or oil seeds, variation in yield per unit N is accounted for by either grain/seed protein concentration or NHI [57,58].

The main problem for grain crops is that the use of crop mass and NNI as cofactors is of reduced interest for analysis of the post-anthesis HI and NHI effect on radiation use efficiency (RUE). The theory underlying the N dilution process as described by Equation (4) is limited to the crop vegetative growth period, when plant growth is solely determined by dry matter and the N uptake in leaves and stems. After anthesis, grain filling and yield formation are determined by the remobilization and transfer of C and N compounds to reproductive organs, which leads to a change in the N–W allometry.

**Figure 8:**
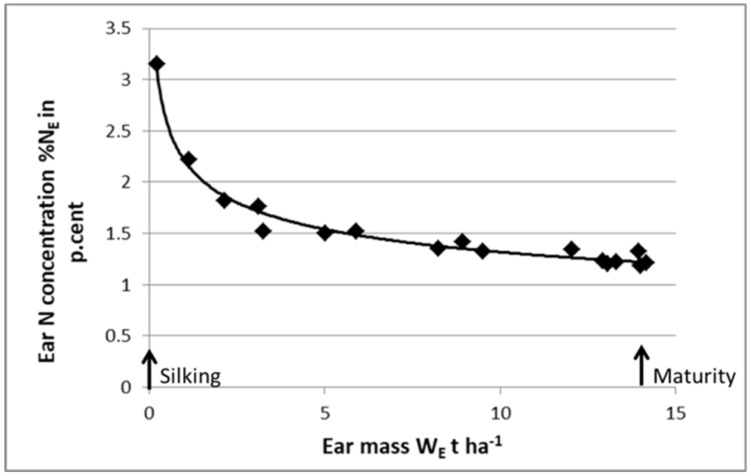
Curve for the critical N dilution in the ear organ for maize crop determined from silking to maturity: %N_E_ = 2.3 W_E_^−025^. %N_E_ = ear N concentration, W_E_ = ear biomass. Data redrawn from Plénet and Lemaire [22].

As shown in Figure 8, it is feasible to define a “critical N dilution curve” for ears of maize during the grain filling period. The ear is considered as a growing organ receiving its C and N resources from the “mother plant”. Then, using this “critical ear curve”, it is possible to perform a post-anthesis diagnosis on crop N nutrition status. Starting with a whole-plant N diagnosis at anthesis, indicating the crop NNI, and then its potential grain number, it would then be possible to perform a second N diagnosis at maturity to determine whether grain filling processes were limited by late N deficiency. Zhao et al. (2020) [59] used this approach for maize and for wheat and demonstrated that the NNI of shoots at anthesis explained grain number, while the NNI of ears at harvest explained grain weight. By using this approach, it should be possible to analyze, in a more comprehensive way, the impact of N nutrition on HI and on the trade-off between yield and grain protein content.

**Figure 9:**
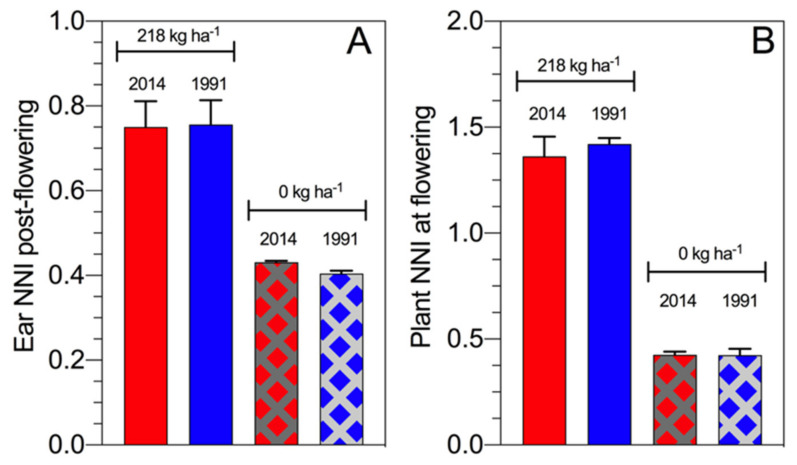
Ear NNI post flowering (including six sampling times during the reproductive period) (panel **A**) and plant NNI at flowering time (panel **B**) for two maize hybrids released in 2014 versus 1991 with full N fertilization and a control (Fernandez and Ciampitti, 2019) [60].

From a historical maize study perspective, the ear NNI post flowering slightly differs between hybrids from the 1990s and 2010s with a similar result for the assessment of the plant NNI at flowering time [60]. Overall, final biomass at maturity values were 28.6 t·ha^−1^ (ear biomass 18.4 t·ha^−1^) for the hybrid from the 2010s and 23.6 t·ha^−1^ (ear biomass 15.2 t·ha^−1^) for the hybrid from the 1990s when full N fertilization was applied, while the difference in biomass without N fertilization was 8.9 t·ha^−1^ (ear biomass 3.7 t·ha^−1^) for the hybrid from the 2010s and 9.2 t·ha^−1^ for the hybrid from the 1991s (ear biomass 3.8 t·ha^−1^) (Figure 9). Therefore, modern maize hybrids maintain the NNI post flowering even when the plant growth and N demand increase over time. Under N deficiency at similar ear biomass, high NNI was reported for the modern maize hybrid relative to the old material, reflecting that the breeding selection process (intentionally or unintentionally) focuses on maize hybrids with a high NNI, primarily during the reproductive period for this crop.

It appears that improving the NRE of crops is the first goal for high NUE in farming systems. This related N efficiency, i.e., the NRE of crops, requires an evaluation under contrasting N supply levels, with low and high N availability. Under low-N conditions, a crop with high NRE would be able to reach its critical N status and then produce its potential yield with a reduced N fertilization supply, which should reduce energy and environmental costs associated with N fertilizer use. Under high-N conditions, when soil N supply is high, a plant with a high NRE would very rapidly exhaust the excess of mineral N in soils, reducing the risk for nitrate leaching and nitrous oxide emissions. As demonstrated, under conditions of low or high N supply, the growth capacity of the crop directly drives its NRE. Therefore, an increase in potential crop growth by both genetic selection and management (e.g., nutrient fertilization, plant density, irrigation) will improve the NRE. Even more intriguing would be an approach to pursue an enhancement in plant N uptake capacity at similar mass, achieving a similar yield with less mineral N fertilization. As a consequence, increasing the plant competitive ability, via a more efficient root system (enhanced length density) for utilizing soil N, needs to be further investigated for different crop species. Although complex, breeding selection for optimizing root architecture to improve soil N acquisition has already been demonstrated. From a management standpoint, investigations of soil improvements via tillage (or no tillage) and soil structure preservation should be focused on improving root health and development with the aim of improving the ability of the plant to take up N, enhancing crop nutrient recovery efficiency.

## 6. Interactions with Water Deficit

Water and N interact strongly in most agricultural conditions with a decrease in water use efficiency (WUE) caused by N deficit and a reciprocal limitation in NUE under water deficit [61,62,63]. The reduction in N uptake under water deficit has been well documented for different species: perennial ryegrass [64], rice [65], wheat [66], and maize [67]. By using the critical N dilution concept for N status diagnosis of perennial grass swards, it was shown that water deficit caused N deficiency despite large N fertilizer application rates [66]. The reduction in N uptake caused by water deficit is the result of two processes: (i) the reduction in crop N demand linked to the reduction in crop mass accumulation due to plant water stress, and (ii) the reduction in soil N availability associated with soil water restriction [68]. As a consequence, the drought-induced reduction in N availability results in a reduction in the crop NNI [69]. Therefore, comparison of NUE across species and cultivars must be done for similar crop water status conditions. For sorghum, it was shown that the crop can maintain its N uptake capacity with a minor effect of water stress with an NNI level close to 1, while maize under the same stress situation presented an NNI level below 0.7 [70]. As a consequence, maize appears to have reached a lower water use efficiency (WUE) in water deficit as compared to irrigated conditions, while sorghum maintained a similar WUE in both situations. The difference between the two species is not due to an intrinsic difference in their response to water deficit per se, but to a difference in their N uptake capacity in dry soil conditions [70]. This aptitude of sorghum to maintain high N uptake capacity in dry soil conditions is linked to its high root density.

To unravel water–nitrogen interactions in field conditions where these two limiting factors are very often acting simultaneously, Kunrath et al. [69] on grassland species and alfalfa and Kunrath et al. [70] on maize and sorghum expressed N uptake in relation to the quantity of water transpired by crop (T) using the ratio N/T. They clearly showed that drought resulted in a lower N/T, indicating that water deficit decreased N uptake in greater proportions than T, and that the decline in N/T was strictly proportional to the decline in transpiration efficiency, i.e., the crop biomass (kg·ha^−1^) product per unit of transpiration water (mm). Consequently, the NUE of different crops has to be compared in similar water conditions and reciprocally, the WUE of different crops must be compared for a similar crop N nutrition status in order to have a functional meaning.

## 7. Old versus New Paradigm for Comparing Changes in N Status and NUE for Crops

**Figure 10:**
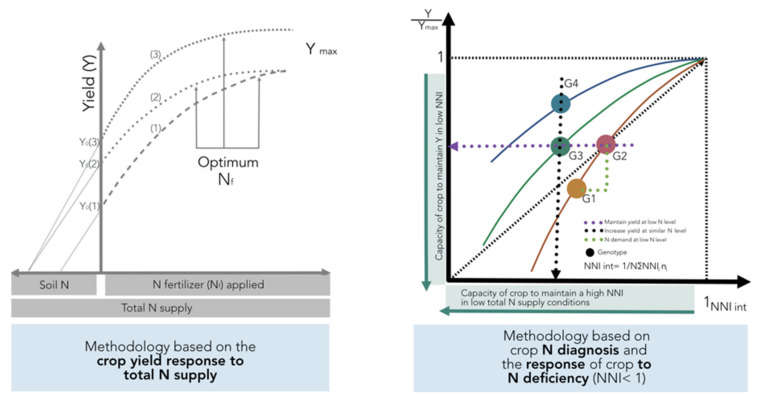
Response of crop yield (Y) to N fertilizer application rates (N_f_) according to variations in soil N supply (Ns) (presented in Figure 1, left panel, methodology using crop yield response to total N supply), relative yield (to the maximum yield in each environment), and integrated N nutrition index (NNI, calculated as the NNI obtained at different growth stages of the crop; a methodology using crop N diagnosis and the response of the crop to N deficiency). Note: for the new approach, comparisons using yield should be done at equal values of harvest index (HI); if HI differs among genotypes, then the best parameter to evaluate is not yield but whole-plant biomass.

As previously defined in Figure 1, variations in NUE among hybrids should be done under a similar range of total N supply in order to understand the contribution of the factors defined by Moll et al. [13] for both soil (NRE) and plant (NCE) processes. If comparisons are not done for a similar range of total N supply, changes in this component and in biomass can mislead the calculation of potential gains in NUE and their components when comparing changes among genotypes. This methodology is based on the crop yield response to total N supply (left panel, Figure 10). In addition, the effect of the environment as related to yield productivity differences should also be taken into account when comparing changes in NUE across G × E × M combinations.

A new method is proposed to account for changes in both yield and N status in order to compare changes in NUE and their components among different genotypes (Figure 10). This improved approach is based on adjusting individual yield to the maximum in each tested environment and for calculating an integrated NNI (NNI int) as the weighted mean of NNI during the crop growth period [14,71]. The main limitation is that one determination of NNI is not sufficient to obtain an adequate picture of the overall N status of the crop during the growth period. Therefore, determination of NNI at critical points during the vegetative and early reproductive periods can facilitate the calculation of the NNI int parameter. For example, for maize, Plénet and Cruz [72] showed that the NNI int (calculated from the early vegetative stage until 20 days after flowering) had a higher correlation with kernel number than kernel weight, strongly influencing final yield. This improved approach permits an evaluation of different scenarios: (i) an increase in relative yield (Y/Ymax) as the NNI int improves for a certain comparison of genotypes (G1 vs. G2; Figure 10), with a capacity to match their own N demand in situations of low N supply (with better NRE), (ii) a similar relative yield with a reduced NNI int, portraying the capacity of a genotype (relative to a counterpart) to maintain yield despite a low N status (G2 vs. G3), and (iii) a greater relative yield under a similar N status, with better NCE for a genotype (G4 vs. G3) (Figure 10, right panel). This new approach should consider a comparison among genotypes at similar HI levels; if HI values differ, then the best term to compare genetic materials will be the overall crop biomass (biomass to maximum biomass level), thereby accounting for potential differences coming from the variation in HI.

In summary, this new improved approach can provide an unbiased assessment of changes in yield and NUE (and their components, NRE and NCE) avoiding the confounding effects of differences in plant N uptake cause by changes in plant biomass and the high uncertainty presented in the traditional method of crop yield response to N supply, relying on two unknown and hard-to-predict components related to soil N supply and the Ymax (crop N demand dependent on the plant growth).

## 8. Conclusions

This review clearly demonstrates the relationship of crop N uptake and its coregulation with (i) soil N supply and (ii) plant growth rate capacity, as well as the necessity to establish bridges between “plant physiology and metabolism” and “field crop ecophysiology”. In addition, NUE strongly depends on the N status of the crop as defined by the NNI and its potential plant growth capacity. This current review provides evidence of changes in NUE related to crop mass and, potentially, in some species related to a genetic change in N uptake efficiency per se when comparing comparable mass levels. Additionally, when considering other resource inputs such as water, comparisons of NUE across species and cultivars must be done in similar crop water status conditions. In summary, this study presents an improved approach that can provide an unbiased assessment of changes in yield and NUE (and their components, NRE and NCE). Future comparisons of changes in NUE (or any plant N-related trait) and other resource use efficiency factors such as WUE (colimitation of N and water) concerning G × E × M interactions can benefit from using the new improved framework to avoid confounding effects of changes in crop mass and NNI. Additionally, the utilization of this new framework will assist researchers in reducing the uncertainty when predicting real changes in NUE relative to the old traditional method of crop yield response to N supply.

## Abbreviations

N	nitrogen
C	carbon
NUE	N use efficiency
NNI	N nutrition index
NRE	N recovery efficiency
Nf	N fertilizer
NCE	N conversion efficiency
HI	harvest index
NHI	N harvest index
%N_c_	critical plant N concentration
Nc	critical N uptake
Wc	maximum biomass without N limitation
WUE	water use efficiency
RUE	radiation use efficiency
T	transpiration
G × E × M	genotype × environment × management
